# The Adequate Number of Histopathology Cross-sections of Temporal Artery Biopsy in Establishing the Diagnosis of Giant Cell Arteritis

**DOI:** 10.18502/jovr.v16i1.8253

**Published:** 2021-01-20

**Authors:** Roshanak Ali-Akbar Navahi, Samira Chaibakhsh, Sayyed Amirpooya Alemzadeh, Kaveh Abri Aghdam

**Affiliations:** ^1^Eye Research Center, The Five Senses Institute, Rassoul Akram Hospital, Iran University of Medical Sciences, Tehran, Iran

**Keywords:** Giant Cell Arteritis, Histopathology Cross-sections, Temporal Artery Biopsy

## Abstract

**Purpose:**

To determine the appropriate number of histopathological cross-sections that are required for a conclusive diagnosis of giant cell arteritis (GCA).

**Methods:**

In this cross-sectional study, the number of sections per slide for paraffin-embedded blocks for 100 randomly selected cases where GCA was suspected and those for negative temporal artery biopsies (TABs) were compared with the number of cross-sections per specimen for eight positive-TABs. All aforementioned examinations were conducted at our center from 2012 to 2016. Then, negative-TABs were retrieved and re-evaluated using light microscopy considering the histopathological findings of GCA.

**Results:**

Ninety-five paraffin blocks were retrieved. The original mean biopsy length was 15.39 ± 7.56 mm. Comparison of the mean number of cross-sections per specimen for both the positive- and negative-TABs (9.25 ± 3.37 and 9.53 ± 2.46) showed that 9.87 ± 2.77 [95% confidence intervals (CI)] cross-sections per specimen were sufficient for a precise GCA diagnosis. There was no statistically significant difference in the mean biopsy length (*P* = 0.142) among the eight positive-TABs. Similarly, no significant difference was observed in the number of cross-sections per specimen (*P* = 0.990) for positive-TABs compared to those for the negative-TABs. After the retrieval of negative-TABs, the mean number of total pre- and post-retrieval cross-sections per specimen was 17.66 ± 4.43. Among all retrieved specimens, only one case (0.01%) showed the histopathological features of healed arteritis.

**Conclusion:**

Positive-TABs did not reveal more histological cross-sections than the negative ones and increasing the number of cross-sections did not enhance the accuracy of TAB.

##  INTRODUCTION

Giant cell arteritis (GCA) is characterized by granulomatous vasculitis of large and medium-sized vessels, and its worldwide annual incidence rate ranges from 1.28 to 29.1 per 100,000 among individuals aged over 50 years.^[1,2,3]^ Approximately 15–20% of GCA patients may develop permanent loss of vision.^[4]^ As per the guidelines of the American College of Rheumatologists (ACR), diagnosis of GCA is primarily based on the presence of characteristic clinical features and laboratory findings of elevated levels of acute-phase reactants.^[5,6,7]^ Temporal artery biopsy (TAB) is considered as the gold standard diagnostic test for GCA.^[8,9]^ A positive-TAB test is mainly defined as vasculitis with infiltration of mononuclear cells with or without the presence of multinucleated giant cells, disruption of the internal elastic lamina, and intimal hyperplasia.^[10,11,12]^ However, sometimes TAB may indicate intermediate findings that make it difficult to distinguish GCA from other pathologies such as healed arteritis or even arteriosclerosis that occurs in elderly patients.^[13,14]^ Thus, TAB has low sensitivity and it may show negative results in 15–40% of patients.^[15,16,17,18,19]^ Additionally, the number of biopsies, length of the artery sampled, sectioning techniques, and histopathological criteria for diagnosing arteritis, presence of skip lesions, and previous treatment with corticosteroids may contribute to false-negative results.^[20,21]^

This study was performed at a tertiary referral center to determine the appropriate number of cross-sections for a TAB examination that are required for a conclusive GCA diagnosis.

##  METHODS

**Figure 1:**
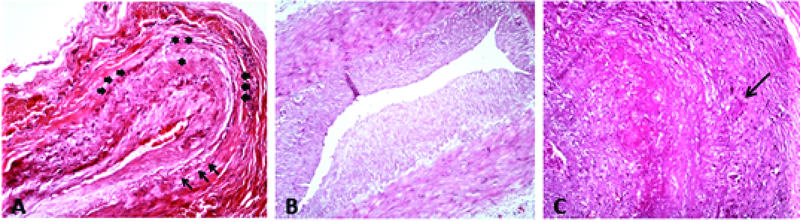
(A) Healed arteritis: note the narrow lumen and minimal intramural lymphocytic infiltration, scarring, and fibrosis (asterisks) in areas with destroyed elastic lamina (short arrows) compared to the areas of intact elastic lamina (long arrows), (H&E staining ×40). (B) Normal artery, negative for GCA (H&E staining ×40). (C) Active GCA: note the obstruction of the lumen, arterial wall thickening, elastic lamina fragmentation, and intramural inflammation with multinucleated giant cells (arrow), (H&E staining ×100). GCA, giant cell arteritis; H&E, Hematoxylin and Eosin

In our center, TAB cross-sections are routinely cut into 2–3 mm-long slices and each of them is embedded transversely in a paraffin block. Next, hematoxylin and eosin-stained serial sections of 5-μm thickness are prepared at three-step levels with 25-μm intervals. TAB specimens are considered positive if a narrow lumen, irregular intimal thickening, and fragmentation of the internal elastic lamina with inflammation of the vessel wall (composed of lymphocytes and epithelioid histiocytes with or without multinucleated giant cells) are observed. In borderline cases including those wherein inflammation is limited to the adventitia, additional levels are requested. In this cross-sectional study, the histopathology reports of 205 archived temporal artery biopsies (TABs; performed between 2012 and 2016) were re-evaluated. The length of the biopsy and total number of cross-sections per specimen for eight positive-TAB cases were compared with those for a 100 computer-assisted randomly selected negative-TABs, which were performed during the same period. Then, paraffin-embedded blocks of these original negative-TABs were retrieved and >90% of each paraffin block was sectioned. A single ophthalmic pathologist (RAAN) re-evaluated all the newly retrieved sections, considering the previously mentioned histopathological findings that characterize GCA. The methods and main outcomes of the study have been summarized in Figure 1. In addition, the revised ACR-2016 (rACR) scores from the available medical records of patients with positive- and negative-TABs conducted in 2016 were evaluated.

**Figure 2:**
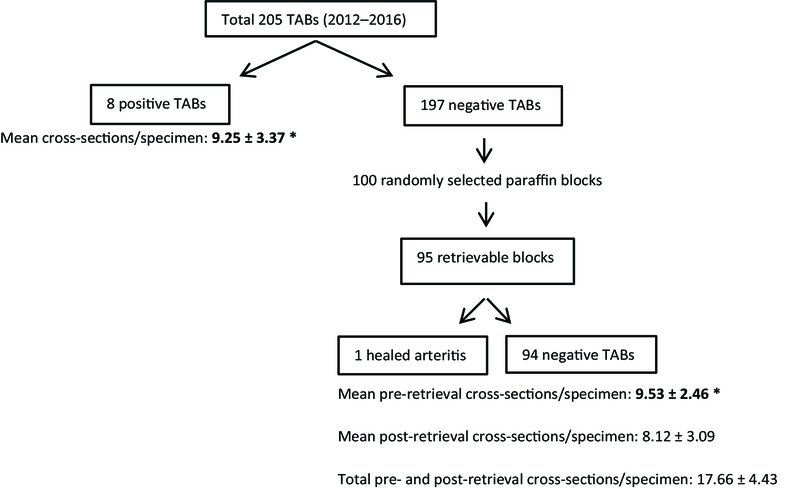
Summary of the methods and main outcomes of the study. *There was no significant difference in the mean number of cross-sections per specimen between the positive- and original negative-TABs (*P* = 0.990). Based on the comparison of these two items, 9.87 ± 2.77 (95% confidence intervals) cross-sections per specimen were considered sufficient for precise results. In addition, retrieval of the original negative-TABs at multiple levels did not enhance the accuracy of TAB for diagnosing GCA. GCA, giant cell arteritis; TAB, temporal artery biopsy

SPSS software version 22.0 (IBM Corp., Armonk, NY) was used for statistical analyses. Results are reported as mean ± standard deviation. The Mann–Whitney U test was used to analyze quantitative variables. *P*-value < 0.05 was considered statistically significant.

##  RESULTS

Of the total 205 TABs conducted during 2012–2016, eight reports were positive for GCA. From the remaining 197 negative biopsies, initially a 100 paraffin-embedded blocks were randomly selected for retrieval. Since five paraffin blocks were not suitable for retrieval, finally the results of 95 specimens were evaluated.

The mean age of the patients was 62.75 ± 12.83 years and 54% were female. Two patients had non-simultaneous bilateral biopsies. The mean biopsy length was 15.39 ± 7.56 mm.

The number of slides per specimen, cross-sections per slide, and the number of slides per mm of biopsy length before and after retrieval have been summarized in Table 1.

**Table 1:** Comparison of positive- and negative-TABs (pre- and post-retrieval)


**Parameter**	**Negative-TAB (** ***N*** ** = 95)**		**Positive-TAB (** ***N*** ** = 8) (original)**	**** ***P*** **-value (between positive-TABs and pre-retrieval negative-TABs)**
	**Pre-retrieval (original)**	**Post-retrieval**	
Mean biopsy length (mm)	15.39 ± 7.56	N/A	16.70 ± 8.48	*P* = 0.142
Mean number of slides/specimen	3.24 ± 0.74	2.83 ± 0.96	3.25 ± 0.82	N/A
Mean number of cross-sections/slide	2.93 ± 0.26	2.84 ± 0.39	2.87 ± 0.36	N/A
Mean number of cross-sections/specimen	9.53 ± 2.46	8.12 ± 3.09	9.25 ± 3.37	*P* = 0.990*
Mean number of cross-sections/mm biopsy length	0.72 ± 0.29	N/A	0.55 ± 0.46	N/A
Mean number of total pre- and post-retrieval cross-sections/specimen	17.66 ± 4.43		N/A	N/A
*Comparisons of positive-TABs and pre-retrieval negative-TABs N/A, not applicable; TAB, temporal artery biopsy				

In the eight positive-TAB specimens, the mean artery length was 16.70 ± 8.48 mm and the mean number of cross-sections per specimen was 9.25 ± 3.37. No statistically significant differences were found in the biopsy length (*P* = 0.142) and the number of cross-sections per specimen (*P* = 0.990) among the eight positive-TABs and when the positive TABs were compared to the pre-retrieval negative-TABs (Table 1). Comparison of the number of cross-sections per specimen for pre-retrieval negative-TABs (9.53 ± 2.46) and those for the eight positive-TABs (9.25 ± 3.37) showed that 9.87 ± 2.77 [95% confidence intervals (CI): 9.16–10.59] cross-sections per specimen were sufficient for precise diagnostic results.

In the clinical evaluation of 95 negative-TABs, we only found 50 cases with complete medical records that met the 2016 rACR criteria,^[7]^ and the mean overall rACR score for these patients was 3.86 ± 1.12. In contrast, the mean overall rACR score for the eight patients with positive TABs in our study was 5.87.

Histopathological evaluation of retrieved biopsies revealed only one case (0.01%) of healed arteritis with mild intramural lymphocytic infiltration, narrowing of the lumen, fragmentation, and destruction of the internal elastic lamina with scarring of the artery wall (Figure 1). This patient had an rACR score of 3, and had undergone bilateral TAB, with original pathology reports showing negative results for GCA.

##  DISCUSSION

Currently, no specific guidelines have been formulated regarding the adequate number of cross-sections needed for accurate biopsy results of TAB specimens.

Although TAB is considered as the gold standard test for diagnosing GCA, ambiguous findings may lead to inconclusive diagnosis or inaccurate results.^[8,9]^ The extent of sectioning, length of the artery, and presence of skip lesions as well as unilateral or bilateral biopsies are among the factors that may affect TAB results.

Characteristic histopathological findings of active GCA include pan-arteritis that is most pronounced in media, with or without multinucleated giant cells and fragmented internal elastic lamina. In contrast, healed arteritis is characterized by diffuse intimal thickening, intimal and medial fibrosis with variable degree of lymphocytic infiltration, loss of internal elastic lamina, and adventitial scarring which correlates with prior history of GCA symptoms and a higher-than-normal erythrocyte sedimentation rate (ESR). Increased ESR is part of the reparative process and not considered a marker for active arteritis.^[22]^ However, occasionally, it may be difficult to distinguish the aforementioned pathology from changes resulting from aging and atherosclerosis.^[23,24,25]^

According to the literature, routine evaluation of TABs at multiple levels does not enhance the diagnostic yield and is not cost-effective.^[20,26,27,28,29]^ In a study conducted by Taylor *et al* ^[29]^ for determining the threshold specimen length for pathological examination and interpretation, there was no statistically significant difference between the number of total cross-sections per specimen used for positive-TABs (22.3) and those for the negative ones (21.6). In our study, there was no statistically significant difference in the mean biopsy length and mean number of cross-sections per specimen for the eight positive-TABs compared to those of the negative-TABs before retrieval. These results indicate that diagnosis in positive-TAB cases did not require a greater number of cross-sections than those required in negative ones.

Methods for the technical processing of a temporal artery differ across centers. Some centers examine the artery in one longitudinal section and two transverse ones, which may be obtained from either end of the artery if the arterial length is sufficient.^[20,29,30]^ TAB processing at our center is performed using transverse sections according to a recommended protocol,^[31]^ with some modifications that have been described in the Methods section.

In this study, we determined that 9.87 ± 2.77 cross-sections per specimen were sufficient to achieve precise results at our center. Further, additional retrieval of the negative-TAB specimens did not increase the chances of obtaining positive GCA results. However, additional studies are required to determine the appropriate number of cross-sections for a TAB evaluation.

“Skip lesions,” which are foci of discontinuous vasculitis, are found in 8–28% of GCA-positive biopsies.^[23,26,32]^ Skip lesions are not common in temporal arteritis, and skipped areas are approximately 330 μm to 1 mm in length.^[27]^ Although the idea is controversial, it has been suggested that a length of 5–7 mm could be the threshold for diagnostic sensitivity of TAB.^[27,33]^ This implies that even short TAB specimens might be sufficient to visualize the histological features of arteritis.^[27]^ Our results indicate that there was only one case of healed arteritis among 95 negative-TAB cases. These results are compatible with those of Chakrabarty *et al*,^[20]^ wherein only 1 out of 132 cases showed positive GCA features after performing sections at multiple levels. However, the length of the artery in our positive case was 13 mm. The extent of the agreement between the first and second slide readings using the Kappa coefficient before and after the retrieval of the negative-TAB specimens could not be calculated due to high similarity between the results. However, regardless of statistical significance, there was approximately a 98% agreement between the two readings since 94 out of 95 negative-TAB specimens were also negative in the second histopathological evaluation.

In general, it is standard to perform a unilateral TAB when GCA is clinically suspected; the contralateral artery biopsy is done if the clinical suspicion is high and the first biopsy is negative.^[34]^ Otherwise, the chance of a positive second biopsy ranges from 5% to 9%,^[35]^ and if the clinical suspicion is low, a unilateral biopsy is sufficient to rule out the diagnosis. The single biopsy after retrieval that was positive for healed arteritis was that of a left temporal artery from a 67-year-old female, which was taken seven days after a negative-GCA result from the first biopsy of the right artery. She had been treated with intravenous methylprednisolone for three days followed by oral prednisolone at a dose of 1 mg/kg before performing TAB.

In general, for cases where GCA is suspected, immediate treatment with high-dose steroids even before a biopsy is recommended. Since the resolution of inflammatory infiltration is usually slow, the chance of detecting active inflammation is not affected by steroid therapy if the biopsy is performed within two weeks.^[36]^ In our case of healed arteritis after retrieval, the specimen was taken seven days after starting steroid therapy. Therefore, the findings could be due to a previous episode of GCA rather than aging-related arterial changes.

The diagnosis of GCA does not always require a positive-TAB, and approximately 15–40% of patients with GCA are TAB-negative.^[15,16,17,18,19]^ This phenomenon where a high percentage of people who have negative biopsies are diagnosed with GCA has resulted in disagreement among neuro-ophthalmologists and rheumatologists regarding the criteria for GCA. It has been recommended that TAB should be performed only for patients with rACR scores of 3 and 4, since there is higher variability in TAB results for other patients.^[7]^ Among the 95 suspected GCA cases with negative TABs, we reviewed the medical records of 50 patients whose mean overall rACR score was 3.86 ± 1.12. These results were similar to those of Abri Aghdam *et al* ^[37]^ (mean score of 3.88 ± 1.19 for negative biopsies). In addition, the mean overall rACR score of the eight patients with positive-TABs in our study was 5.87. After retrieval of negative-TABs, we identified only one case of healed arteritis with an rACR score of 3.

Positron emission tomography^[38]^ and 3 tesla-magnetic resonance imaging^[39]^ are new technologies that are now being regularly used in the diagnosis and monitoring of GCA disease progression. Although, the use of non-invasive color duplex ultrasonography reduces the chances of false-negative TABs due to skip lesions,^[40]^ it is an operator-dependent technique.

It is important to consider that the final diagnosis in TAB-negative patients may indicate a spectrum of conditions mainly including other rheumatologic diseases, presence of non-temporal arteries with GCA, infectious diseases, neoplastic diseases, and neuro-ophthalmic conditions.^[7,41]^

In conclusion, positive-TABs in our study did not require more cross-sections than the negative ones. Further, TAB examination at multiple levels did not increase the diagnostic yield of the test. In this study, 9.87 ± 2.77 cross-sections per specimen were sufficient for a precise diagnosis of GCA.

##  Financial Support and Sponsorship

This work was funded by the Eye Research Center of Iran University of Medical Sciences which did not have any role in the design, execution, and presentation of results.

##  Conflicts of Interest

There are no conflicts of interest.
